# Systemic Mastocytosis: A Mimicker of Reactive Arthritis

**DOI:** 10.1155/2023/6655005

**Published:** 2023-08-07

**Authors:** Oussama G. Nasrallah, Razan Mohty, Jean El-Cheikh, Mira Merashli

**Affiliations:** ^1^Division of Urology, Department of Surgery, American University of Beirut Medical Center, Beirut, Lebanon; ^2^Division of Hematology, Oncology, Department of Internal Medicine, American University of Beirut Medical Center, Beirut, Lebanon; ^3^Division of Rheumatology, Department of Internal Medicine, American University of Beirut Medical Center, Beirut, Lebanon

## Abstract

**Objectives:**

Illustration of a case of systemic mastocytosis mimicking reactive arthritis in the absence of an infectious etiology.

**Methods:**

Review of the patient's medical records.

**Results:**

We report a case of systemic mastocytosis relapse, presenting with pancytopenia accompanied by knee monoarthritis, cystitis, and bilateral conjunctivitis occurring simultaneously at the same time interval within 2–4 days, mimicking reactive arthritis in the absence of an infectious etiology.

**Conclusion:**

Our case demonstrated reactive arthritis features (triad of urethritis, conjunctivitis, and arthritis) without an infectious trigger but rather a relapse of mastocytosis. We should think outside the box when faced with such a clinical scenario in the absence of an infectious etiology. Paraneoplastic reactive arthritis is to be considered after excluding an underlying infection.

## 1. Introduction

Systemic mastocytosis is characterized by over proliferation and production of mast cells and their infiltration in various tissues, leading to numerous clinical presentations [1]. Systemic mastocytosis may initially present with nonspecific symptoms such as fatigue, nausea, and diarrhea, which may later evolve into pruritus, flushing, itching, and anaphylaxis. It may also present as gastrointestinal bleeding and various neuropsychiatric symptoms [2]. However, with the involvement of the bone marrow, anemia, leukopenia, and thrombocytopenia become appreciated upon laboratory investigations.

Reactive arthritis (ReA) belongs to the family of spondyloarthropathies associated with gastrointestinal and genitourinary symptoms which typically appear secondary to an infectious process [3]. It typically presents with the classic triad of arthritis, conjunctivitis, and urethritis; however, most patients may not necessarily present with the full-triad of symptoms.

In our case, we illustrate the case of a 48-year-old lady presenting with an exacerbation of her systemic mastocytosis with knee monoarthritis, cystitis, and bilateral conjunctivitis, thereby mimicking reactive arthritis. To our knowledge, this is the first case published in the literature of systemic mastocytosis manifesting with such a triad of knee monoarthritis, conjunctivitis, and cystitis.

## 2. Case Description

We report a case of a 48-year-old lady known to have systemic mastocytosis who was in clinical remission for 6 months, presenting with a 2-day history of right knee pain, swelling, and difficulty walking; a 4-day history of dysuria and suprapubic tenderness; a 3-day history of bilateral conjunctivitis described as eye redness and pain upon blinking; and a persistent fever of 39 degrees Celsius 1 day prior to presentation. She denied any respiratory or gastrointestinal symptoms in the past few months. The patient reported no sexual activity for more than a year and no recurrent oral or genital ulcers. She denied any travel history in the past 25 years. Blood tests done in the ER showed pancytopenia that was not present 2 weeks prior to her presentation, as shown in Table 1.

The diagnosis of systemic mastocytosis was based on the bone marrow findings summarized in Table 1 in addition to the cKIT D816V gene mutation that was found at diagnosis one year ago.

Initially, she had received 6 months of cladribine, a purine nucleoside analogue, for curative intent, achieving complete remission after 7 cycles with no evidence of residual disease on repeated bone marrow biopsy. She was then switched to midostaurin, a tyrosine kinase inhibitor targeting, among others, the KIT D816V mutation, at a dose of 100 mg twice daily as a bridge for haploidentical stem cell transplantation (haplo-SCT) from her brother. The patient could not tolerate midostaurin due to nausea and vomiting, leading to the discontinuation of midostaurin one week prior to her presentation to the emergency department.

In the emergency room, she was started empirically on piperacillin/tazobactam 4.5 mg every 8 hours after the full-infectious workup was done (Table 1). Notably, her labs showed anemia, thrombocytopenia, and eosinophilia, denoting a possible disease progression.

Fifteen milliliter of semiturbid synovial fluid were aspirated from the right knee joint and sent for gram stain and culture before the administration of antibiotics, and further analyses were done on the sample during her admission (Table 1).

The entire infectious workup was negative including the synovial fluid, blood, and urine cultures, and the 16S ribosomal RNA in the synovial fluid was negative as well.

After excluding an infectious etiology, steroids were initiated: methylprednisolone 1 mg/kg per day, resulting in marked improvement within 24 hours: she was able to ambulate her right knee with minimal pain and swelling with resolution of the dysuria and conjunctivitis. The following day, she was restarted on midostaurin, antiemetic, continued methylprednisone, and then discharged on a tapering regimen for methylprednisone. The patient improved clinically; her complete blood count showed an increase in hemoglobin and platelet counts, and her symptoms did not recur; therefore, it showed a response to treatment.

Review of her medications, including cladribine and midostaurin, did not show any association with her presenting symptoms. Midostaurin was found to be associated with urinary tract infections and reactive joint arthritis; however, our patient did not have any infection evidence, and she stopped the medication a couple of days prior to the onset of her symptoms.

The patient improved clinically and received haplo-SCT later on with no complications. She is currently in remission post-haplo-SCT.

## 3. Discussion

Mastocytosis is a constellation of disorders characterized by an overproliferation and production of mast cells and their infiltration in various tissues, leading to numerous clinical presentations [1]. Systemic mastocytosis can present as fatigue, pruritus, flushing, itching, nausea, diarrhea, gastrointestinal bleeding, various neuropsychiatric symptoms, and anaphylaxis [2]. When the bone marrow is involved, a depression in hematopoietic lineages may be seen such as anemia, leukopenia, and thrombocytopenia, as seen in our patient.

The diagnosis of SM (systemic mastocytosis) is made based on major and minor criteria including a bone marrow and/or affected organ biopsy that would reveal the extent of involvement of the disease. Specific mutations have been identified and implicated in the pathogenesis of SM [4].

Reactive arthritis (ReA) is typically associated with a previous infection by bacterial pathogens such as *Chlamydia trachomatis*, *Salmonella* spp., *Shigella*, *Campylobacter jejuni*, and *Yersinia enterocolitica* [3]. It typically presents with the classic triad of arthritis, conjunctivitis, and urethritis; however, most patients may not necessarily present with the full-triad of symptoms. Arthritis usually affects the lower extremities (which may be monoarticular or polyarticular) and sacroiliac joints. In addition, urethritis, balanitis, and sterile pyuria often present as urological manifestation [5].

ReA is usually more common in patients who are HLA-B27 positive; however, not all patients with ReA are HLA-B27 positive. Approximately 50% of patients are HLA-B27 positive [3, 5]. The prevalence of HLA-B27 in all spondyloarthropathies in Lebanon is 13.87% which is much lower than the worldwide population where prevalence exceeds 90% in ankylosing spondyloarthritis [6]. Our patient did not get tested for HLA-B27 due to financial reasons of not being covered by the insurance company in the setting of its low prevalence in Lebanon. She also did not complain of inflammatory back pain.

Our patient did not have any infectious etiologies: viral workup was negative, indicating that the dysuria cannot be explained by a viral etiology. As for the bacterial etiologies, the patient was sexually inactive long before her symptoms started. In addition, she did not suffer from any gastrointestinal symptoms prior to her presentation, and all cultures including blood, urine, and synovial fluid were all negative. The 16S ribosomal RNA in the synovial fluid was also negative. Procalcitonin was on the low side. Based on all these information, we believe that the bacterial etiology is highly unlikely as well. Moreover, these symptoms started around 1 week after stopping the midostaurin. As for the infiltration of the synovial fluid, it showed no bacteria in the synovial fluid analysis or significant infiltration by neutrophils which would indicate bacterial infection. The patient improved significantly after 1 day of the initiation of methylprednisolone. In additional, the urine analysis and culture were both negative, and the dysuria resolved after the initiation of methylprednisolone. Finally, the conjunctivitis characterized by redness in color and pain upon blinking resolved without additional intervention. The speed of resolution of these symptoms with the use of methylprednisolone is probably because the patient had paraneoplastic ReA triggered by the mastocytosis and not by infection, which explains the response to the dose of steroids given. To our knowledge, this is the first case in literature addressing such an entity.

Treatment of ReA focuses on abolishing the main etiology which is usually infectious prior to the development of symptoms. The etiologies include infections such as *Chlamydia trachomatis*, *Salmonella* spp., *Shigella*, *Campylobacter jejuni*, and *Yersinia enterocolitica* infections which have triggered the symptoms [7]. As for the rheumatological symptoms, the use of nonsteroidal anti-inflammatory agents (NSAIDs) is the first line of treatment in most cases; systemic steroid therapy may be used when NSAIDs fail to provide complete relief of symptoms [8]. Our patient was treated with steroids with gradual tapering followed by midostaurin and haplo-SCT. NSAIDs were not tried first since her symptoms were intense with marked fatigue, so we thought that steroids would be a better option in her case.

The exact mechanism by which mastocytosis could possibly trigger ReA is unknown and needs to be studied as the data in the literature are lacking. HLA-B27 is assumed to modulate the interaction between the bacteria and immune cells in the usual cases where the infectious etiology is the trigger, but we know very well that only 50% of ReA cases are associated with positive HLA-B27. There should be other genes that predispose to this autoimmune mechanism which triggers ReA.

## 4. Conclusion

Our case demonstrated reactive arthritis features (triad of urethritis, conjunctivitis, and arthritis) without an infectious trigger but rather a relapse of mastocytosis. We should think outside the box when faced with such a clinical scenario in the absence of an infectious etiology. Paraneoplastic reactive arthritis (ReA) is to be considered after excluding an underlying infection.

## Figures and Tables

**Table 1:** Bone marrow biopsy results and laboratory data.

Test	Result		
*Bone marrow biopsy results*			
Flow cytometry	No evidence of acute leukemia or increased blasts		
CD25	Positive		
CD68	Weakly positive		
CD117	Positive		
CD3, CD5, CD10, CD20, CD34, MPO, TDT, cyclin D1, DBA-4A, BCL6	Negative		
Ki67	5%		
Full-leukemia panel for translocation	Negative		
Karyotype	46 XX		
Histology	90% cellular bone marrow Diffuse population of medium-sized cells with round to kidney-shaped nuclei, and moderate to abundant cytoplasm with retraction, seen surrounding aggregates of small lymphocytes and admixed with increased eosinophils		

*Blood test done while on midostaurin 2 weeks prior to presentation*:			
CBC	Hb: 10.3 g/dL MCV: 103.7 fL WBCs: 4100/*µ*L	Neutrophils: 70% Lymphocytes: 21%	Eosinophils: 4% Platelets: 117000/*µ*L

*Blood test in the ED on presentation*			
CBC	Hb: 7.8 g/dL MCV: 104.2 fL WBCs: 3500/*µ*L	Neutrophils: 74% Lymphocytes: 15%	Eosinophils: 3% Platelets: 75000/*µ*L
CRP	172 mg/dL		
ESR	119 mm/h		
Procalcitonin	0.45 ng/mL		

*Urine studies in the ED on presentation*			
Urine analysis	WBCs: numerous; RBCs: 2–4 Leukocyte esterase: negative; nitrites: negative; squamous epithelia: present casts: negative; crystals: negative; bacteria: negative		
Urine culture	Negative		

*Infectious workup during admission*			
PCR (parvovirus, adenovirus, chlamydia, gonorrhea, TB)	Not detected		
Bacterial culture	Negative		
Fungal culture	Negative		
AFB smear	Negative		
16S RNA	Negative		
Synovial fluid analysis	RBCs: 5610/*µ*L; WBCs: 1230/*µ*L; *N*: 77%; *L*: 12%; Mono: 8%; Eo: 3%		
Synovial fluid polarizing microscopy	No crystals seen Many RBCs and 10–15 WBCs		

ED: emergency department; CBC: complete blood count; CRP: C-reactive protein; ESR: erythrocyte sedimentation rate; WBCs: white blood cells; RBCs: red blood cells; PCR: polymerase chain reaction; TB: tuberculosis; AFB: acid fast bacilli. *N*: neutrophils; *L*: lymphocytes; Mono: monocytes; Eo: eosinophils.

## Data Availability

The data reported in our case report can be retrieved from the patient's chart at the American University of Beirut Medical Center upon request if needed.
